# Temporal dysregulation of *PPARG-PRKAG2* co-expression in gray matter: Implications for cognitive decline and intervention targets in type 2 diabetes

**DOI:** 10.21203/rs.3.rs-7907793/v1

**Published:** 2025-10-22

**Authors:** Shelli R. Kesler, Kimberly A. Lewis, Heather Cuevas, Elena Flowers

**Affiliations:** University of Texas at Austin; University of California Los Angeles; University of Texas at Austin; University of California

**Keywords:** type 2 diabetes, cognitive decline, imaging transcriptomics, MRI, clinical dementia rating, miRNA-20b, metformin

## Abstract

**Background:**

Type 2 diabetes mellitus (T2DM) is associated with increased risk for cognitive decline and diagnosis of Alzheimer’s disease (AD). The mechanisms of T2DM related dementia remain unclear.

**Methods:**

Brain magnetic resonance imaging was retrospectively obtained for 1,802 adults (age 66 +/− 9 years, 47% male) of whom N = 271 had T2DM. We applied an accelerated longitudinal design and imaging transcriptomics to non-invasively examine the group-level trajectories of *PPARG*and *PRKAG2* co-expression in gray matter.

**Results:**

Gene expression trajectories differed significantly between T2DM and controls (χ^2^ = 13.82, p = 0.001). Co-expression was higher in early stages and then weakened in later stages among T2DM, while remaining stable over time in controls. *PPARG* and *PRKAG2* co-expression was significantly associated with cognitive function in controls (F = 3.17, p < 0.001) but not T2DM (F = 7.72, p = 0.299) suggesting dysregulated or failed compensatory mechanisms. Individuals with T2DM not taking metformin demonstrated unstable gene co-expression over time compared to those taking metformin (χ^2^ = 12.42, p = 0.006).

**Conclusions:**

The convergence of *PPARG*-mediated metabolic remodeling and *PRKAG2*/AMPK-driven energy sensing may act as a coordinated neuroprotective mechanism, upregulated in response to cellular stress in both pathological (T2DM) and normative (aging) contexts. However, these processes appear to become dysregulated in T2DM, potentially resulting in cognitive decline and increased risk for dementia.

## Background

Individuals with type 2 diabetes (T2DM) are at significantly increased risk for neurodegeneration and associated cognitive decline, including higher incidence of Alzheimer’s disease (AD). Potential mechanisms for T2DM-related neurodegeneration include metabolic dysfunction, neuroinflammation, cerebrovascular disease, disrupted gut microbiome, and insulin resistance (1, 2). T2DM and AD also share several common molecular risk factors involved in metabolism, insulin signaling, inflammation, and toxin clearance (3–5). Many of the genes that regulate these processes are also critical for neuronal development and health.

Using non-invasive imaging transcriptomics, we previously demonstrated that genes involved in insulin signaling, glucose metabolism (*IRS1, AKT1, PPARG, PRKAG2*) and neurotransmission (*GRIN2B*) were significantly expressed in regions of T2DM-related gray matter atrophy (6). Gene expression patterns interacted in both T2DM and non-diabetic control groups to predict cognitive function. However, these patterns were significantly altered in T2DM, with *PPARG* (peroxisome proliferator-activated receptor gamma) taking over normal directional pathways from other genes to influence cognitive function. Specifically, higher *PPARG* was associated with higher *PRKAG2* (protein kinase AMP-activated non-catalytic subunit gamma 2) expression which in turn was associated with lower Clinical Dementia Rating (CDR) in T2DM (i.e., +PPARG ◊ +PRKAG2 ◊ −CDR). Given that this was a significant difference in gene network structure compared to controls, increased *PPARG* influence may represent a compensatory mechanism in the context of T2DM neuropathology. The alteration in gene pathways occurred despite relatively preserved neuropsychological function, equivalent amyloid burden compared to controls, and lower apolipoprotein allele 4 (*APOE4*) risk. Thus, these molecular changes may represent an early compensatory change in T2DM (6).

However, our prior study was cross-sectional, and although we utilized Bayesian network analysis which determines a directional pathway model analogous to structural equation modeling, our findings were fundamentally associative. We therefore conducted a follow-up study that applied an accelerated longitudinal design in a larger sample. This approach allows for modeling temporal trajectories by combining cross-sectional data from different cohorts, each observed at a different point in time. Although each participant was observed only once, time was treated as a continuous variable, and individuals were evaluated across timepoints to approximate a longitudinal pattern. This method provides insight into group-level temporal effects, but not individual change. Thus, this represents a proof-of-concept study to support further research.

We again utilized imaging transcriptomics to non-invasively evaluate gene expression patterns associated with T2DM neuropathology. Neuroimaging data is spatially co-localized with gene expression patterns from transcriptomic brain atlases to facilitate the study of in vivo molecular mechanisms underlying brain health and disease (7–10). By linking gene expression patterns directly to brain structure and function, imaging transcriptomics opens new avenues for exploring the molecular mechanisms of neurological diseases and for developing targeted therapies. Several imaging transcriptomics studies have been conducted in neurodegenerative diseases (11–13) but very few have involved T2DM (14–17). Based on our prior findings, we hypothesized that co-expression of *PPARG* and *PRKAG2* would change over time in T2DM compared to controls. We expected that T2DM would display early compensatory activation followed by later failure consistent with prior evidence regarding neurologic compensation in neurodegeneration (18).

## Methods

### Participants

We obtained data from the Mayo Clinic Study of Aging (MCSA) via the Laboratory of Neuroimaging’s Image and Data Archive at the University of Southern California. The MCSA is a prospective, epidemiologic study of cognitive aging that began in 2004 in Olmsted County, Minnesota (19). The Mayo Clinic and Olmsted Medical Center institutional review boards approved the parent study from which this data was extracted and gave approval for the sharing of anonymized data. All participants provided written informed consent during the parent study. For the current study, privacy rights of participants were observed, and no personally identifiable information was obtained. The MCSA is a longitudinal study, but only cross-sectional data is currently available in the Image and Data Archive. However, participants in the cross-sectional cohort were assessed at different time points (i.e., no repeated measures; cross-sectional data at multiple timepoints from different individuals). Thus, these data lend themselves well to an accelerated longitudinal design for approximating a longitudinal trajectory across individuals. Visits were separated by approximately 1 year across 10 years. Table 1 indicates the sample sizes at each Visit. Given that visits 10 and 11 had only one participant’s data available, these visits were excluded from the analyses.

During the MCSA, participants underwent a medical history review, neurological evaluation, and provided demographic information. Trained nurses reviewed medical records to obtain data on chronic conditions including diagnosis of T2DM (20, 21). At the time of our study, there were N = 22,239 cases in the Image and Data Archive, but only 1,802 had magnetic resonance imaging (MRI) data and N = 271 of these had T2DM. During the MCSA, participants were administered several neurological and neuropsychological assessments. For this study, we examined data from the CDR given our prior results (6). Additionally, participants underwent genotyping to determine *APOE4* status (positive or negative).

*Gray Matter Volume Measurement.* Anatomic brain MRIs were performed on all consenting individuals with no contraindications. Those who underwent MRI were not significantly different demographically from those who did not, per previous report (22). Imaging data were collected with a 3 Tesla GE scanner (GE Medical Systems, Milwaukee, WI) using a 3D magnetization prepared rapid acquisition gradient echo sequence (repetition time = 2300 ms, echo time = 3 ms, inversion time = 900 ms, flip angle = 8°, field of view = 26 cm, in-plane matrix = 256 × 256, phase field of view = 0.94, slice thickness = 1.2 mm).

We measured gray matter volumes from anatomic brain MRI using voxel-based morphometry implemented in Statistical Parametric Mapping (SPM) 12 (Wellcome Trust Centre for Neuroimaging, London, UK) (23). Successful segmentation and normalization were confirmed using visual and quantitative quality assurance methods. We restricted gray matter measurement to regions of atrophy that were observed in our prior study.

#### Imaging Transcriptomics.

We obtained brain transcriptome data from the Allen Human Brain Atlas (AHBA), which is currently considered the most comprehensive transcriptional brain map available (7). AHBA was developed using six adult human donor brains to provide expression data from tens of thousands of genes measured from thousands of brain regions (24). To determine the co-localization of brain imaging and transcription data, we used the Multimodal Environment for Neuroimaging and Genomic Analysis toolbox v3.1 (25) to correlate gray matter volume with patterns of AHBA expression from the *PPARG* and *PRKAG2*. Principal Components Analysis was performed on a region-by-gene expression matrix to identify components explaining at least 95% variance across the AHBA donors. The component scores were then entered as independent variables into a multiple linear regression analysis with the corresponding gray matter volume values as the dependent variable. The accuracy of the probe to gene mapping was verified and probe data were normalized to z-scores. Representative probes were then selected in a data-driven manner considering between-donor homogeneity and the distributions of probe data. Genomic autocorrelations were calculated using an intraclass correlation coefficient (ICC) to measure the gene expression reliability across donors.

#### Statistical Analyses.

Demographics were compared between groups at each visit using ANOVA or Chi squared tests, as appropriate. R squared values for *PPARG* and *PRKAG2* were extracted for each participant. These values represent the strength of the image-transcriptome co-localization. As shown in Table 1, data density decreased substantially after visit 5, but restricting analyses to visits 1–4 would have excluded later stages of the trajectory and biased inferences toward earlier phases. Thus, to evaluate the co-expression trajectory of *PPARG* and *PRKAG2* across study visits, we used generalized additive models (GAMs), which are well-suited for irregular or sparse longitudinal data (26–28). GAMs stabilize estimates at timepoints with limited observations by borrowing strength from neighboring values through smooth basis functions, while still allowing for localized deviations where sufficient data exist. To prevent overfitting, particularly in sparsely sampled later visits, we used a basis dimension parameter (k = 5), which controls the complexity of the smooth and acts as a regularization term.

We evaluated whether the relationship between visit and *PPARG* expression in predicting *PRKAG2* differed between groups by comparing two GAMs: one with a shared smooth surface for visit across all participants (joint-smooth model) and another with separate smooths stratified by T2DM group (stratified-smooth model). Both models included fixed effects for T2DM status, a smooth term for age, and linear terms for biological sex and body mass index (BMI). Models were compared using an approximate likelihood ratio test, given that GAMs use penalized likelihood. We additionally reported the change in Akaike Information Criterion (AIC) to aid interpretation.

We conducted a supplemental analysis using a traditional linear regression model to further support the GAM findings given the sparse data in later visits. We divided the cohort into visit stages with “early” stage defined as visits 1–2 (1039 controls, 175 T2DM) and “late” stage (396 controls, 96 T2DM) as Visits 3 and higher. This division was based on the results from the GAM (see below for details), which indicated that in T2DM, the strongest co-expression between *PPARG* and *PRKAG2* occurred at Visits 1 and 2. At Visit 3, the marginal effect of *PPARG* on *PRKAG2* weakened. The linear regression modeled *PRKAG2* expression from the interaction between *PPARG*, visit stage, and T2DM status (i.e., *PPARG* × visit stage × T2DM) with age, biological sex, and BMI as additional covariates.

To examine whether the co-expression of *PPARG* and *PRKAG2* was associated with cognitive function and whether this relationship differed by T2DM status, we fitted a GAM with CDR sum as the dependent variable. The model included a group-stratified bivariate smooth interaction between *PPARG* and *PRKAG2* expression, allowing the gene-gene association to vary flexibly between T2DM and controls. Covariates included T2DM group, a smooth term for visit to capture nonlinear temporal trends, a smooth term for age, and linear terms for biological sex and BMI.

Finally, we compared *PRKAG2* expression in the T2DM group between those who were or were not taking metformin. The GAM included *PPARG*, metformin (yes/no), age, sex, and BMI.

Data analyses were conducted in the R Statistical Package (v4.5.1, R Foundation, Vienna, Austria). GAM models were conducted using the “mgcv” library with restricted maximum likelihood for primary models and maximum likelihood for model comparisons. Alpha level for all analyses was p < 0.05.

## Results

Mean age across groups was 65.94 +/− 8.55 years at visit 1 and 85.37 +/−2.3 years at visit 9 representing a 20-year age span across approximately 9 years of assessments (Table 1). There were no significant group-by-visit effects in terms of age (F = 1.001, p = 0.428), education (F = 1.45, p = 0.169), sex (χ^2^ = 10.46, p = 0.234), or *APOE4* status (χ^2^ = 3.18, p = 0.074). BMI was higher in the T2DM group during earlier visits (F = 2.56, p = 0.009).

**Figure 1:**
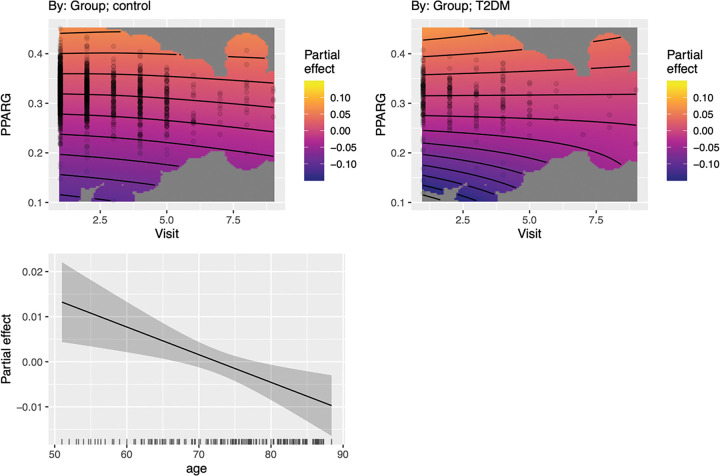
*PRKAG2*Expression Across Visits. T2DM and controls demonstrated significantly different trajectories of *PRKAG2* expression over time (χ^2^ = 13.82, p = 0.001). Top panels: In T2DM, *PPARG*was strongly associated with higher *PRKAG2* expression during early visits, but this relationship diminished in later visits. In controls, the *PPARG–PRKAG2*relationship was more stable across time. Each surface was adjusted for covariates. Contour lines represent constant predicted values of *PRKAG2* across combinations of visit and *PPARG*expression, based on the fitted GAM surface. Curved lines indicate a strong interaction effect between *PPARG*and visit, and tighter spaced lines indicate a rapid change in *PRKAG2* levels. Bottom panel: partial effects of smoothed covariates. The solid line indicates the estimated smooth function with the shaded area representing the 95% confidence interval.

*PRKAG2* expression changed significantly over time for T2DM (F = 69.99, p < 0.001) and controls (F = 270.23, p < 0.001). The difference in trajectories was significant (χ^2^ = 13.82, p = 0.001, Δ AIC = −9.93). Male sex (t = 2.49, p = 0.013) and smoothed age (F = 16.71, p < 0.001) were significant contributors to the model. In T2DM, *PPARG* strongly predicted higher *PRKAG2* in early stages. This effect weakened or plateaued in later visits. In controls, the relationship between *PPARG* and *PRKAG2* was stable over time (Fig. 1).

The supplemental linear regression model of early vs. late stage supported the GAM results. The *PPARG* × T2DM interaction was significant and positive (estimate = 0.121, p = 0.002) indicating that *PPARG* predicts higher *PRKAG2* in T2DM in the early stage. The *PPARG* × stage × T2DM interaction was significant and negative (estimate = −0.140, p = 0.039) suggesting that the relationship between *PPARG* and *PRKAG2* declines (or reverses) in the late stage. The stage × T2DM interaction was significant and positive (estimate = 0.041, p = 0.048) indicating that individuals with T2DM in the late stage have higher baseline *PRKAG2*, controlling for *PPARG*.

**Figure 2:**
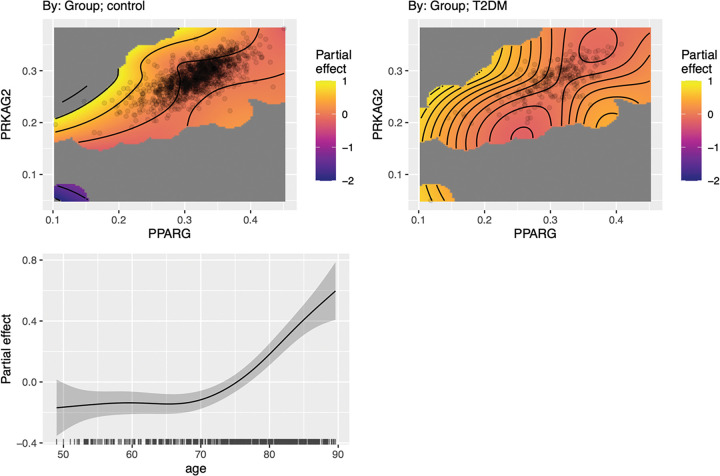
Effect of *PPARG-PRKAG2*Co-expression on CDR. *PPARG*-*PRKAG2*co-expression was significantly associated with CDR in controls (F = 3.17, p < 0.001) but not in T2DM (F = 7.72, p = 0.299). Top panels: contour color gradients represent CDR values (warmer shades = higher predicted impairment). Each surface was adjusted for covariates. The T2DM group showed a nonlinear co-expression pattern suggestive of dysregulated compensatory dynamics, whereas controls showed a more stable, linear surface. Bottom panels: partial effects of smoothed covariates. The solid line indicates the estimated smooth function with the shaded area representing the 95% confidence interval.

*PPARG*-*PRKAG2* co-expression significantly influenced CDR in controls (F = 3.17, p < 0.001) but was not significant for T2DM (F = 7.72, p = 0.299). Visualization of the model (Fig. 2) indicated a non-monotonic (inverted U-shaped) relationship between gene co-expression and CDR in T2DM. Alternatively, controls demonstrated a more stable, linear association with CDR. Age was a significant contributor to the model (F = 16.70, p < 0.001) but sex and BMI were not significant (p > 0.203).

**Figure 3:**
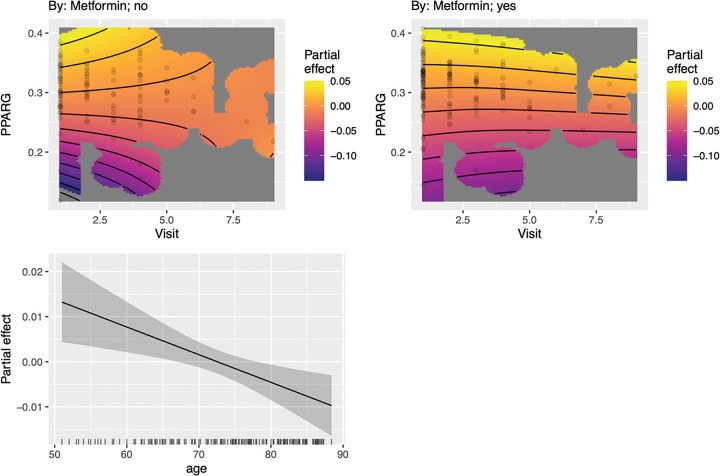
Differences in PPARG-PRKAG2 Co-expression Associated with Metformin. Individuals with T2DM taking metformin demonstrated significantly higher *PRKAG2* expression across visits compared to those who were not taking metformin (t = 2.39, p = 0.018). Expression trajectories differ significantly between the metformin positive and negative groups (χ^2^ = 12.42, p = 0.006). Top panels: *PPARG-PRKAG2* co-expression was stable and consistently positive over time in individuals taking metformin but was heterogeneous across time in those not taking metformin, with shifts between positive and negative partial effects. Each surface was adjusted for covariates. Bottom panels: partial effects of smoothed covariates. The solid line indicates the estimated smooth function with the shaded area representing the 95% confidence interval.

Individuals with T2DM who reported taking metformin demonstrated significantly higher *PRKAG2* expression across visits compared to those who were not taking metformin (t = 2.39, p = 0.018). Expression changed over time in the metformin positive (F = 39.02, p < 0.001) and metformin negative (F = 32.45, p < 0.001) groups and these trajectories were significantly different (χ^2^ = 12.42, p = 0.006, Δ AIC = −4.62). As shown in Fig. 3, gene co-expression was stable and positive for the metformin group but was less stable, fluctuating over time, and showing more variability in direction and magnitude in the metformin negative group.

## Discussion

We previously observed significant alteration in the brain transcriptome of individuals with T2DM compared to controls with a potential compensatory associative pathway from *PPARG* to *PRKAG2* supporting cognitive function (6). In the present study, we aimed to further evaluate this relationship by examining the pseudo-longitudinal trajectory of *PPARG-PRKAG2* co-expression in gray matter tissue and the effect of this trajectory on cognitive function. In T2DM, *PPARG* strongly predicted higher *PRKAG2* in early stages, consistent with a compensatory mechanism. This effect weakened or plateaued in later visits, possibly due to loss of regulatory control or biological exhaustion. In controls, the relationship between *PPARG* and *PRKAG2* was stable over time, likely reflecting normal regulatory function without disease-driven compensation or failure.

This finding was supported by supplemental stage-based modeling which indicated that, in early-stage T2DM, higher *PPARG* was associated with higher *PRKAG2* expression, consistent with compensatory upregulation. In late-stage T2DM, this compensatory relationship reversed, possibly reflecting system failure or overload. These findings are consistent with prior evidence regarding functional compensation in the context of neuropathology. The brain is often capable of reorganization to counteract decline and preserve function. However, ongoing pathology tends to overwhelm these compensatory mechanisms, resulting in functional decline (18).

*PPARG* encodes peroxisome proliferator-activated receptor gamma, a transcription factor central to lipid metabolism, adipogenesis, and insulin sensitivity (29). *PPARG* activation downregulates NF-κB signaling, reducing neuroinflammation, which is a hallmark of both aging and T2DM-associated cognitive decline (30, 31). Additionally, upregulation of *PPARG* may counteract oxidative stress and insulin resistance in the brain (32). *PRKAG2* is a regulatory subunit of AMP-activated protein kinase (AMPK), a master energy sensor that restores energy balance by stimulating catabolic pathways and inhibiting anabolic processes (4). AMPK activation is critical in maintaining ATP levels during metabolic stress, which is common in both aging brains and T2DM (33). *PRKAG2* is also involved in the clearance of damaged proteins and toxins, which accumulate during neurodegeneration. Accordingly, *PRKAG2* expression has been shown to be elevated in AD and is associated with cognitive impairment in T2DM (3, 34).

With aging or disease, neurons experience reduced mitochondrial efficiency, increased oxidative stress, and altered glucose metabolism. A rise in *PPARG–PRKAG2* co-expression may represent a compensatory adaptation with *PPARG* driving transcriptional programs that enhance lipid utilization, and AMPK signaling helping to restore energy balance. Their co-expression could synergistically buffer against metabolic decline in the aging or T2DM brain. We showed that both T2DM and control groups demonstrated increased *PRKAG2* expression over time consistent with normal age-related changes. However, T2DM showed significantly higher *PRKAG2* expression in later stages despite de-coupling with *PPARG*, potentially reflecting dysregulation of compensatory processes or accelerated aging. Prior studies have demonstrated evidence of accelerated cortical brain aging in individuals with T2DM (35, 36) but our findings represent the first evidence of accelerated molecular brain aging.

*PPARG* agonists, especially pioglitazone, show protective actions in experimental models of AD and diabetic encephalopathy (37). In rodent studies, pioglitazone reversed beta-amyloid–induced memory deficits and restored learning functions (38, 39). In human trials involving patients with mild AD or amnestic mild cognitive impairment, pioglitazone improved cognitive scores (40, 41). *PPARG* agonists work in part by reducing amyloid-beta levels, lowering inflammatory and oxidative markers, stabilizing brain-derived neurotrophic factor, and improving measures of insulin sensitivity (42, 43).

However, our findings indicate that upregulation of *PPARG* may shift from adaptive to maladaptive over time in the T2DM brain. Prior research has shown that excessive or prolonged *PPARG* activation is linked to metabolic dysregulation such as overeating and weight gain through neuronal pathways regulating energy balance (44, 45). In our cohort, participants with T2DM initially met criteria for obesity (BMI > 30), but later declined below this threshold, which may reflect early *PPARG* upregulation followed by later downregulation or improved diabetes management. Yet BMI did not contribute significantly to our gene expression or cognitive models, suggesting that *PPARG*-related brain effects may operate independently from adiposity. Because BMI is a coarse measure of metabolic status, factors such as insulin resistance, adipose distribution, inflammation, or diet quality may better reflect *PPARG*-related metabolic variance. Incorporating these refined measures into future studies could clarify how peripheral metabolic remodeling and central *PPARG* signaling interact over time to influence neurocognitive trajectories of T2DM. Further, we did not have access to blood assay data to examine the potential effects of blood glucose level or inflammatory markers on our findings. However, this research emphasizes the need for a balanced homeostatic regulation between neuroprotective and metabolic pathways to sustain cognitive function in T2DM and highlight the importance of metabolic phenotyping beyond BMI.

MicroRNAs regulate mRNA translation and can cross the blood brain barrier, creating the possibility that peripherally administered therapeutic interventions might influence central nervous system function. Our prior study showed that circulating miR-20b is inversely associated with longitudinal changes in fasting glucose (46) and *PPARG* is a functionally validated mRNA target of miR-20b (47). We also showed that circulating miR-20b decreased after two years of treatment with metformin compared to an increase in expression in a placebo group, who had higher rates of incident T2DM (48). Because miR-20b is linked to *PPARG* suppression, this pattern implies that higher miR-20b expression in untreated individuals may reduce *PPARG* expression, theoretically contributing to the increased diabetes risk observed in the placebo group. These observations provide preliminary evidence that microRNAs such as miR-20b may be a therapeutic approach to modulate *PPARG*-related transcriptomic changes associated with neurodegeneration and cognitive decline in T2DM.

*PPARG*–*PRKAG2* co-expression was significantly associated with cognitive function as measured by the CDR in controls but not in T2DM. This dissociation may imply a loss of regulatory control, breakdown of compensation, or uncoupling of biological mechanisms in T2DM. The relationship in T2DM was U-shaped where higher CDR (worse cognition) occurred at both low and high gene co-expression levels, consistent with failed compensation at extremes. These findings may also suggest that alternative neurobiologic processes are recruited to support cognitive function in T2DM. Previous studies suggest that cognitive performance is preserved in T2DM by an increase in functional brain connectivity (49, 50). Functional neuroimaging was not available for this cohort in the Imaging Data Archive and therefore, future studies that include multimodal imaging transcriptomics are required.

Metformin treatment was associated with increased, stable *PRKAG2* expression across visits in the T2DM group. This suggests a sustained engagement of the brain’s AMPK pathway that may contribute to metformin’s reported neuroprotective effects on neuronal metabolic equilibrium during chronic energetic stress (51, 52). This stability suggests that metformin may recalibrate neuronal metabolism toward cognitive resilience, promoting continuous energy balance and proteostatic clearance through mTOR inhibition and autophagy induction (52–54). These findings align with emerging evidence that genetic variability in PRKAG2 influences individual responses to metformin (55) and that metformin helps maintain blood-brain barrier integrity via AMPK activation (56). These observations extend the role of metformin beyond glycemic control, positioning it as a modulator of brain bioenergetic homeostasis that could delay or attenuate the molecular cascades linked to T2DM, cognitive decline, and AD (57). Future mechanistic and translational work should determine whether persistent *PRKAG2* stabilization represents a biomarker of adaptive neural metabolism or a viable target for interventions aimed at preventing diabetes-related neurodegeneration.

There are several limitations to this study. We utilized an accelerated longitudinal design which provides insight into group-level trajectories but not individual changes. True longitudinal studies are needed to evaluate change in brain transcriptome pathways among individuals with T2DM and the impact of these changes on cognitive function and risk for AD. We examined the co-expression of *PPARG* and *PRKAG2* because this was the primary altered pathway related to cognitive function in our initial study of T2DM and to reduce comparisons considering the sample size. However, alteration of other brain transcriptome pathways may also be important for cognitive function in T2DM. Data in later visits were very sparse, and we addressed this using penalized GAMs and a supplementary analysis of early versus late visits. However, analyses may have lacked power to detect alternative trajectories. While the AHBA is the most comprehensive brain transcriptome atlas to date, it was developed based on a small sample. The atlas includes gene expression patterns for thousands of brain regions but does not comprehensively cover the entire brain.

In conclusion, our findings suggest that alterations of *PPARG-PRKAG2* co-expression may be involved in the brain’s response to both normative aging and neuropathological conditions associated with T2DM. However, this response appears to become dysregulated over time in T2DM. Our results provide novel insights regarding the potential molecular mechanisms of T2DM-related cognitive decline and the risk for AD. Further research is required that includes younger adults with T2DM to determine how early these changes occur. Data regarding blood glucose levels would also provide important insights regarding the impact of T2DM disease management on the brain transcriptome. Multimodal neuroimaging will be necessary to further examine functional compensatory changes and to investigate the role of the brain transcriptome in these changes.

## Figures and Tables

**Table 1.** 

Visit	Group	N	Age	Male	BMI	CDR	Education	APOE4	Metformin	PPARG	PRKAG2
1	Control	724	65.47 (8.4)	377 (52%)	28.37 (4.9)	0.103 (0.49)	14.88 (2.5)	216 (30%)	2 (0.28%)	0.325 (0.04)	0.294 (0.03)
	T2DM	121	68.70 (8.9)	70 (58%)	31.62 (5.2)	0.218 (0.74)	13.92 (2.3)	27 (22%)	73 (60%)	0.312 (0.05)	0.288 (0.04)
2	Control	315	68.44 (9.2)	152 (48%)	28.48 (5.0)	0.102 (0.41)	15.03 (2.6)	97 (31%)	1(0.32%)	0.319 (0.04)	0.289 (0.03)
	T2DM	54	71.51 (8.0)	33 (61%)	30.26 (3.7)	0.269 (0.70)	14.61 (2.5)	16 (30%)	32(59%)	0.312 (0.03)	0.286 (0.03)
3	Control	113	73.72 (8.6)	57 (50%)	27.22 (4.6)	0.296 (1.14)	14.92 (2.9)	34 (30%)	0(0%)	0.313 (0.04)	0.283 (0.03)
	T2DM	33	73.64 (8.0)	20 (61%)	33.74 (5.6)	0.273 (0.55)	13.91 (2.7)	8 (24%)	21(64%)	0.298 (0.04)	0.280 (0.03)
4	Control	155	78.98 (5.5)	78 (50%)	27.30 (4.5)	0.386 (1.10)	14.24 (3.0)	43 (28%)	0(0%)	0.306 (0.04)	0.279 (0.03)
	T2DM	38	79.32 (4.8)	28 (74%)	29.10 (5.3)	0.395 (1.16)	14.26 (2.6)	6 (16%)	15(40%)	0.307 (0.05)	0.275 (0.03)
5	Control	83	81.99 (4.0)	48 (58%)	26.82 (4.4)	0.470 (1.28)	14.60 (3.1)	19 (23%)	0(0%)	0.299 (0.04)	0.275 (0.03)
	T2DM	14	81.89 (3.5)	9 (64%)	29.89 (6.8)	0.107 (0.21)	14.57 (2.5)	5 (36%)	8(57%)	0.293 (0.03)	0.269 (0.03)
6	Control	18	81.91 (3.7)	8 (44%)	27.51 (4.9)	0.806 (1.61)	13.78 (2.6)	4 (22%)	0(0%)	0.284 (0.04)	0.273 (0.03)
	T2DM	5	84.16 (3.6)	3 (60%)	29.26 (4.4)	0.900 (125)	12.20 (3.0)	3 (60%)	1(20%)	0.299 (0.03)	0.270 (0.01)
7	Control	8	83.31 (3.6)	3 (37.5%)	27.92 (4.7)	0.188 (0.53)	14.25 (2.6)	4 (50%)	0(0%)	0.288 (0.03)	0.277 (0.03)
	T2DM	1	78.80 (N/A)	1 (100%)	25.77 (N/A)	N/A (N/A)	16.00 (N/A)	1 (100%)	1(100%)	0.237 (N/A)	0.250 (N/A)
8	Control	14	84.74 (2.2)	10 (71%)	26.73 (3.4)	0.214 (0.38)	15.00 (3.5)	4 (29%)	0(0%)	0.314 (0.05)	0.286 (0.03)
	T2DM	3	85.20 (3.4)	1 (33%)	26.80 (2.1)	0.167 (0.29)	10.67 (2.3)	3 (100%)	2(67%)	0.281 (0.03)	0.282 (0.02)
9	Control	5	85.88 (2.0)	5 (100%)	26.11 (2.5)	0.100 (0.22)	16.00 (3.2)	1 (20%)	0(0%)	0.308 (0.02)	0.287 (0.02)
	T2DM	2	84.10 (3.4)	2 (100%)	29.32 (3.1)	0.000 (0.00)	17.00 (4.2)	0 (0%)	1(50%)	0.273 (0.08)	0.280 (0.04)

## Data Availability

The dataset supporting the conclusions of this article is available to qualified researchers and industry partners from the Laboratory of Neuroimaging’s Image and Data Archive at the University of Southern California (http://loni.usc.edu) or the Global Alzheimer’s Association Interactive Network (https://www.gaain.org).

## Abbreviations

T2DM: type 2 diabetes
AD: Alzheimer’s disease
PPARG: Peroxisome Proliferator-Activated Receptor Gamma
PRKAG2: Protein Kinase AMP-Activated Non-Catalytic Subunit Gamma 2
MRI: Magnetic resonance imaging
AHBA: Allen Human Brain Atlas
GAM: Generalized additive model
CDR: Clinical Dementia Rating
BMI: Body mass index
AIC: Akaike Information Criterion
MCSA: Mayo Clinic Study of Aging
AMPK: AMP-activated protein kinase
